# Metabolome- and genome-scale model analyses for engineering of *Aureobasidium pullulans* to enhance polymalic acid and malic acid production from sugarcane molasses

**DOI:** 10.1186/s13068-018-1099-7

**Published:** 2018-04-04

**Authors:** Jun Feng, Jing Yang, Wenwen Yang, Jie Chen, Min Jiang, Xiang Zou

**Affiliations:** 1 College of Pharmaceutical Sciences, Chongqing Engineering Research Center for Pharmaceutical Process and Quality Control, Southwest University, 2 Tian Sheng Road, Beibei, Chongqing, 400715 People’s Republic of China; 2 Wuhan Sunhy Biology Co., Ltd, Wuhan, 430074 People’s Republic of China; 3 School of Chemical Engineering & Pharmacy, Wuhan Institute of Technology, Wuhan, 430205 People’s Republic of China; 4 State Key Laboratory of Materials-Oriented Chemical Engineering, College of Biotechnology and Pharmaceutical Engineering, Nanjing Tech University, Nanjing, 211816 People’s Republic of China

**Keywords:** *Aureobasidium pullulans*, Metabolic engineering, Polymalic acid, Pyruvate carboxylase, Sugarcane molasses

## Abstract

**Background:**

Polymalic acid (PMA) is a water-soluble biopolymer with many attractive properties for food and pharmaceutical applications mainly produced by the yeast-like fungus *Aureobasidium pullulans*. Acid hydrolysis of PMA, resulting in release of the monomer l-malic acid (MA), which is widely used in the food and chemical industry, is a competitive process for producing bio-based platform chemicals.

**Results:**

In this study, the production of PMA and MA from sucrose and sugarcane molasses by *A. pullulans* was studied in shake flasks and bioreactors. Comparative metabolome analysis of sucrose- and glucose-based fermentation identified 81 intracellular metabolites and demonstrated that pyruvate from the glycolysis pathway may be a key metabolite affecting PMA synthesis. In silico simulation of a genome-scale metabolic model (*i*ZX637) further verified that pyruvate carboxylase (*pyc*) via the reductive tricarboxylic acid cycle strengthened carbon flux for PMA synthesis. Therefore, an engineered strain, FJ-PYC, was constructed by overexpressing the *pyc* gene, which increased the PMA titer by 15.1% compared with that from the wild-type strain in a 5-L stirred-tank fermentor. Sugarcane molasses can be used as an economical substrate without any pretreatment or nutrient supplementation. Using fed-batch fermentation of FJ-PYC, we obtained the highest PMA titers (81.5, 94.2 g/L of MA after hydrolysis) in 140 h with a corresponding MA yield of 0.62 g/g and productivity of 0.67 g/L h.

**Conclusions:**

We showed that integrated metabolome- and genome-scale model analyses were an effective approach for engineering the metabolic node for PMA synthesis, and also developed an economical and green process for PMA and MA production from renewable biomass feedstocks.

**Electronic supplementary material:**

The online version of this article (10.1186/s13068-018-1099-7) contains supplementary material, which is available to authorized users.

## Background

Polymalic acid (PMA) is a water-soluble biopolymer composed of l-malic acid (MA) monomers and is mainly produced by the yeast-like fungus *Aureobasidium pullulans* [1]. PMA has free carboxyl groups, making it simple to perform chemical modifications and create various derivatives or carrier-linked pro-drugs. Due to its unique properties, including high water solubility, biocompatibility, and biodegradability, PMA has attracted an increasing attention as a drug carrier or biomaterial in the past few years and is expected to have applications in the preparation of various polymeric micelles, microparticles, nanoconjugates, and nanoparticles for drug delivery systems [2–5]. PMA also has the potential to be used as a nano-imaging agent for safe and noninvasive diagnosis in the clinical setting [6]. In addition, its monomer MA can be easily generated from PMA via acid hydrolysis. Like other dicarboxylic acids, MA is also an important organic acid and is regarded as a C4 platform chemical in the food and pharmaceutical industries, for which there is a strong global market of over 600,000 tons/year, with an annual growth rate of 4% [7, 8].

Inexpensive renewable feedstocks and media for fermentation, as well as high PMA titers with good productivity are also important constraints in developing industrial PMA fermentation processes. The previous studies have demonstrated fermentative production of PMA from low-cost substrates, such as sweet potato [9], corncob [10, 11], soy molasses [12], and wheat straw [13]. However, starchy and lignocellulosic feedstocks must first be subjected to thermochemical, dilute acid, or enzymatic treatments to release fermentable sugars; these limitations must be overcome to achieve an economically competitive process, including enhanced strain tolerance and fermentation performance in the presence of inhibitors released from dilute acid-pretreated lignocellulose [8].

In this study, we aimed to develop an economical and environmentally friendly bioprocess for PMA and MA production from sugarcane molasses as a feedstock. Sugarcane molasses is the main by-product of sugar production and contains ~ 50% reducing sugars, mainly sucrose and some glucose and fructose; in addition, sugarcane molasses can be used directly in fermentation without requiring any pretreatment [14, 15], and its price is ~ 40% lower than corn (starch and dextrose) based on the fermentable sugar content according to the current market prices. Therefore, sugarcane molasses is considered a relatively inexpensive and renewable biomass feedstocks for biorefinery applications due to its rich sugar content and cost-effectiveness. As omics analysis developing rapidly over the last decade, metabolome analysis has been employed to characterize intracellular metabolic states for understanding cell metabolism and improving the production of target metabolites [16]. Metabolomics has been a powerful tool for understanding the intracellular metabolism with broad range of applications in various fields, including medical science [17], synthetic biology [18], medicine [19], plant systems [20], and microbial systems [21]. In this study, we evaluated the performance of the strain *Aureobasidium pullulans* for the utilization of different sugars, including sucrose, fructose, and glucose contained in sugarcane molasses, and compared the metabolic processes of sucrose- and glucose-based fermentation through metabolome analysis. In silico simulation of a genome-scale model of *A. pullulans* was further verified, and the metabolic node containing pyruvate carboxylase via the reductive tricarboxylic acid (TCA) cycle was engineered to enhance PMA production. Finally, direct sugarcane molasses fermentation, without any pretreatment or nutrient supplementation, was developed for PMA production. This work will be beneficial for the development of an economical and green process for PMA and bio-based MA production from renewable biomass feedstocks.

## Methods

### Strains, media, and culture conditions

The strain *A. pullulans* CCTCC M2012223 was isolated by our laboratory and can be obtained from the China Center for Type Culture Collection (Wuhan, China). This strain was maintained on potato dextrose agar slants. The seed culture medium contained 60-g/L glucose, 2-g/L NH_4_NO_3_, 0.1-g/L KH_2_PO_4_, 0.1-g/L MgSO_4_, 0.1-g/L ZnSO_4_, 0.5-g/L KCl, and 20-g/L CaCO_3_. The seed culture was grown in a 500-mL shake flask containing 50-mL liquid medium and incubated at 25 °C on a rotary shaker (180 rpm) for 2 days. The fermentation medium contained 90-g/L sugar (glucose, sucrose, xylose, arabinose, or fructose), 2-g/L NH_4_NO_3_, 0.1-g/L KH_2_PO_4_, 0.1-g/L MgSO_4_, 0.1-g/L ZnSO_4_, 0.5-g/L KCl, and 20-g/L CaCO_3_. *Escherichia coli* DH5α cells were employed for routine DNA manipulations. *E. coli* was grown in LB medium (5 g/L yeast extract, 10 g/L tryptone, 10 g/L NaCl, pH 7.0) at 37 °C, and kanamycin or ampicillin (50 mg/L) was added when required. *Agrobacterium tumefaciens* AGL1 was grown on YEB medium (10-g/L yeast extract, 5-g/L tryptone, 5-g/L sucrose, 0.5-g/L MgSO_4_·7H_2_O, pH 7.0) at 28 °C, and kanamycin or carbenicillin (50 mg/L) was added when required.

### Sampling, quenching, and extraction of intracellular metabolites

The cells of strain *A. pullulans* CCTCC M2012223 were collected after culturing for 48 and 72 h with glucose- and sucrose-based fermentation, respectively, and intracellular metabolism was quenched by immediately adding five volumes of prechilled 60% (v/v) methanol. After quenching at − 40 °C, cells were pelleted in a centrifuge (4000×*g*, 4 °C, 10 min). The pellets were washed with phosphate-buffered saline (pH 7.4), frozen in liquid nitrogen, and stored at − 80 °C. Next, 60 mg per sample with six biological replicates was ball-milled into a fine powder under frozen conditions with 1 mL of extraction mix consisting of deionized water (50%), methanol (50%), and the internal standards nonadecanoic acid (0.2 mg/mL) and isotope alanine (10 mM), and then lyophilized. The samples were derivatized by vortexing with 60 μL of 15-mg/mL methoxypyridine hydrochloride for 30 s and then incubating for 2 h at 37 °C. The samples were then trimethylsilylated by adding 60-μL bis(trimethylsilyl) trifluoroacetamide (BSTFA) with 1% trimethylchlorosilane and incubating for 90 min at 37 °C. Subsequently, an extract containing all intracellular metabolites was collected via centrifugation at 12,000×*g* for 10 min at 4 °C [22].

### Metabolite detection with gas chromatography–mass spectrometry (GC–MS)

GC–MS analysis was performed to detect the metabolites in the samples. Briefly, 1 μL sample was injected into an Agilent 7890A/5975C GC–MS system (Agilent Technologies, CA, USA) with a 20:1 split injection ratio. The system was equipped with an HP-5MS capillary column (5% phenyl methyl silox: 30 m × 250 μm i.d., 0.25 μm; Agilent J&W Scientific, Folsom, CA, USA). The inlet temperature was maintained at 280 °C, the interface temperature was 150 °C, and the ion source temperature was 250 °C. The GC temperature program was as follows: hold at 70 °C for 2 min, ramp at 10 °C/min to 300 °C, and a final hold at 300 °C for 5 min. Helium was used as carrier gas at a constant flow rate of 1 mL/min, with a total run time of 30 min. MS conditions were as follows: electron ionization (EI); full-scan mode (*m/z* 35–780); electron energy, 70 eV [23]. The chromatographic differences between sample groups were obvious, and the retention time was reproducible and stable, demonstrating the reliability of metabolomics analysis.

### Data processing and statistical analysis

XCMS software was used to identify and quantify mass spectral peaks. Data preprocessing, including raw data filtering, peak detection, alignment, normalization, and identification, was performed automatically. Metabolites were identified by comparison of mass spectra with the National Institution of Standards and Technology (NIST), Wiley metabolome, Golm metabolome (GM), and in-house databases (Additional file 1: Table S1, red color) [24]. Six independent experiments were performed for each conclusion. Pattern recognition methods based on principal component analysis (PCA) and orthogonal-partial least squares-discriminant analysis (OPLS-DA) were then carried out using SIMCA-P V13.0 (Umetrics AB, Umea, Sweden). All variables were unit variance scaled before PCA and OPLS-DA. Hierarchical clustering analysis was carried out using R software (version 3.1.3) to visualize and group metabolite profiles. A list of variance metabolites that contributed mostly to the model was generated from the *P* values obtained using Student’s *t* tests (*P* < 0.05) and variable importance in the projection values (> 1). In addition, the false discovery rate (FDR) significance criterion was performed with an FDR limit of 0.05 to avoid false-positive results in analyzing differential metabolite correlations. Metabolic pathways were constructed according to pathway analysis of the Metaboanalyst and KEGG metabolic databases [25, 26].

### In silico analysis of a genome-scale metabolic model

The reconstructed genome-scale metabolic network (*i*ZX637) of *A. pullulans*, containing 1347 reactions and 1133 metabolites, was used for constraint-based flux analyses [27] to determine the most effective route for maximizing PMA production and minimizing the influence of biomass accumulation. The synthesis of PMA is closely related to MA synthesis in *A. pullulans*. Therefore, three routes of MA synthesis, the oxidative branches of the TCA cycle, the reductive branches of the TCA cycle, and the glyoxylate shunt, were examined. In silico, constrained reactions of the oxidative branches of the TCA cycle contained oxoglutarate dehydrogenase, aconitate hydratase, fumarase, succinate dehydrogenase, and succinate-CoA ligase. Constrained reactions of the reductive branches of the TCA cycle contained malate dehydrogenase, and constrained reactions of the glyoxylate shunt contained fumarase, isocitrate lyase, and glyoxylate lyase. For in silico simulation, the reaction fluxes corresponding to one route of MA synthesis (the oxidative branches of the TCA cycle, the reductive branches of the TCA cycle, or the glyoxylate shunt) were constrained from 0 to 140 mmol/gDW/h (the reaction fluxes of other two routes of MA synthesis were set to 0 gDW/h), and the cell growth rate was constrained from 0 to 1 gDW/h. The specific PMA synthesis rate was then maximized as an objective function and the substrate uptake rates for glucose in the simulation were constrained by 10 mmol/gDW/h. The specific PMA synthesis rate at different flux values of the cell growth rate and route of MA synthesis were plotted on the *Z*-axis of the flux profile graph. In addition, because the reductive branches of the TCA cycle were assumed to be located only in the cytoplasm in silico, the flux of malate dehydrogenase located in the mitochondria was set to 0 mmol/gDW/h in simulation of three routes. The basic tools used for the model analysis were flux balance analysis (FBA) [28]. GLPK (freeware) (http://www.gnu.org/software/glpk/) was used as a linear programming solver. The COBRA Toolbox 2.0 [29] was used for calculation and analysis in the MATLAB environment.

### Gene cloning and expression in the host strain

The DNA of *A. pullulans* was extracted using a TIANamp Yeast DNA Kit (TIANGEN, China) and was used as a template to amplify the pyruvate carboxylase (*pyc*, EC: 6.4.1.1) gene. First, one 3.748-kb *pyc* fragment was amplified using *Sma*I-*pyc*-S and *Mun*I-*pyc*-A, as listed in Additional file 1: Table S1. Second, the *pyc* fragment obtained was double digested with *Sma*I and *Mun*I and then ligated into plasmid pBARGPE1, which was also treated with *Sma*I and *EcoR*I (*Mun*I isocaudarner) to generate the plasmid pBARGPE1-*pyc*, which contained a gpdA promoter (P*gpdA*) and a trpC terminator (T*trpC*) flanking the *pyc* gene. The P*gpdA*-*pyc*-T*trpC* cassette was amplified by primers P*gpdA*.*pyc*.T*trpC*-S and P*gpdA*.*pyc*.T*trpC*-A. The P*gpdA*-*pyc*-T*trpC* cassette was cloned into the *EcoR*I site of the pK2-P*trpC*-*hyg*-T*trpC* plasmid using a One Step Cloning Kit (Vazyme, USA), forming the plasmid pK2-*hyg*-*pyc* for *A. tumefaciens*-mediated transformation (ATMT), as described in our previous study [30]. T-DNA insertion sites were analyzed using a Genome Walking Kit (TaKaRa, Japan) in ATMT-derived clones. Polymerase chain reaction (PCR) primers were used to amplify the T-DNA flanking sequence (Additional file 1: Table S1).

### Batch and fed-batch fermentation in a 5-L stirred-tank fermentor

Batch fermentation kinetics were studied in a 5-L stirred-tank fermentor (Shanghai Baoxing Co., Ltd., China) containing 3.5 L of fermentation media (with glucose as carbon resource) or only diluted sugarcane molasses. The fermentation medium was inoculated with 350 mL of the seed culture grown in a shake flask for 48 h and operated at 25 °C with agitation and aeration at 400–700 rpm and 0.8 vvm, respectively. During fermentation, the agitation was controlled to maintain the dissolved oxygen level at greater than 20%. For sugarcane molasses fermentation, the crude sugarcane molasses (containing ~ 420 g/kg of total sugar and 14.29 ± 0.27 g/kg of total nitrogen) were diluted by water to ~ 90 g/L total sugar. For fed-batch fermentation, when the total sugar concentration was lower than 30 g/L, sugarcane molasses (approximately 270-g/L reducing sugars) was continuously added to maintain the total sugar concentration at approximately 10–20 g/L. Broth samples were collected periodically for the analysis of residual sugar, biomass, and PMA titers. All trials were performed in triplicate.

### Quantitative reverse transcription (RT)-PCR

Total RNA was extracted using a Fungal RNA Kit (Omega, USA) and reverse transcribed into cDNA using reverse transcriptase (TIANGEN). The quantitative PCR assay was performed using the SYBR Green method (FastQuant RT Super Mix; TIANGEN). Primers for the *pyc* gene are shown in Additional file 1: Table S1. The gene encoding β-actin was used as the reference gene.

### Analytical methods

Biomass was determined by the dry cell weight method. Prior to measurement, excessive CaCO_3_ was eliminated from the broth by adding 1-M HCl. The cell suspension was centrifuged at 4000×*g* and then dried overnight at 105 °C [31]. PMA was analyzed by centrifuging the fermentation broth and the resulting supernatant was mixed with an equal volume of 2-M H_2_SO_4_ in an incubator at 85 °C for 8 h. The hydrolyzed PMA sample was analyzed by high-performance liquid chromatography (Agilent 1260, USA) using a Spursil C18-EP organic acid column at 40 °C and eluted with 5-mM H_2_SO_4_ at a rate of 0.6 mL/min to determine its malic acid content [32]. The glucose concentration was measured using a fully automatic residual sugar analyzer (Biology Institute of Shandong Academy of Sciences, China).

## Results and discussion

### Evaluation of carbon sources in shake-flask culture

**Fig. 1:**
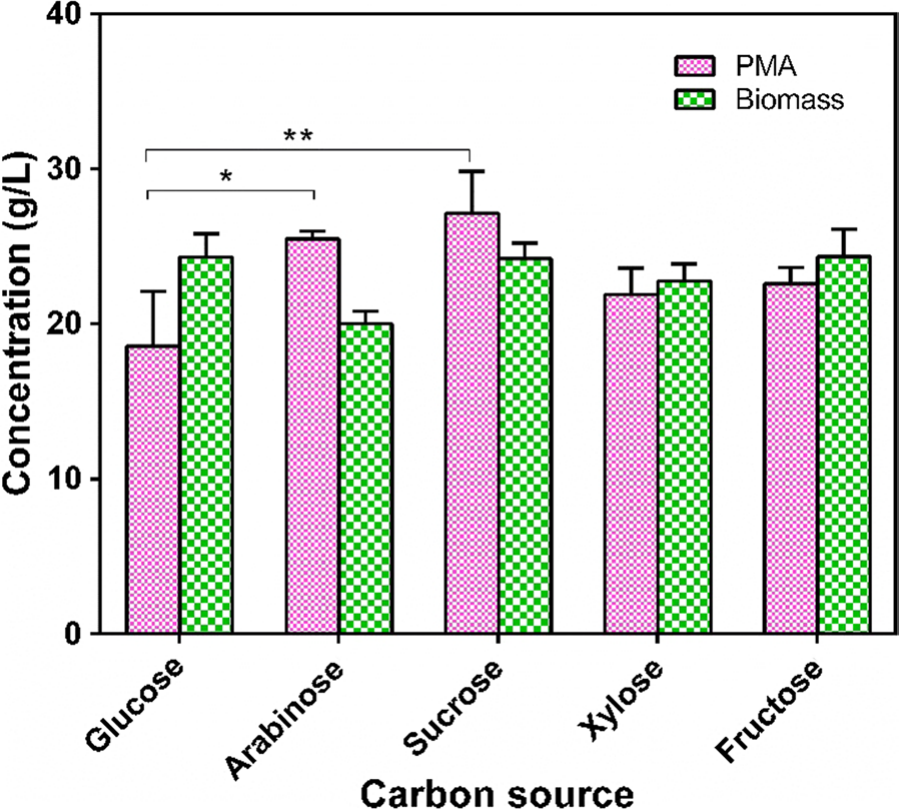
Effects of carbon sources on cell growth and PMA production in shake-flask. Values are the means and standard deviations of three independent experiments. *P < 0.05, **P < 0.01

**Fig. 2:**
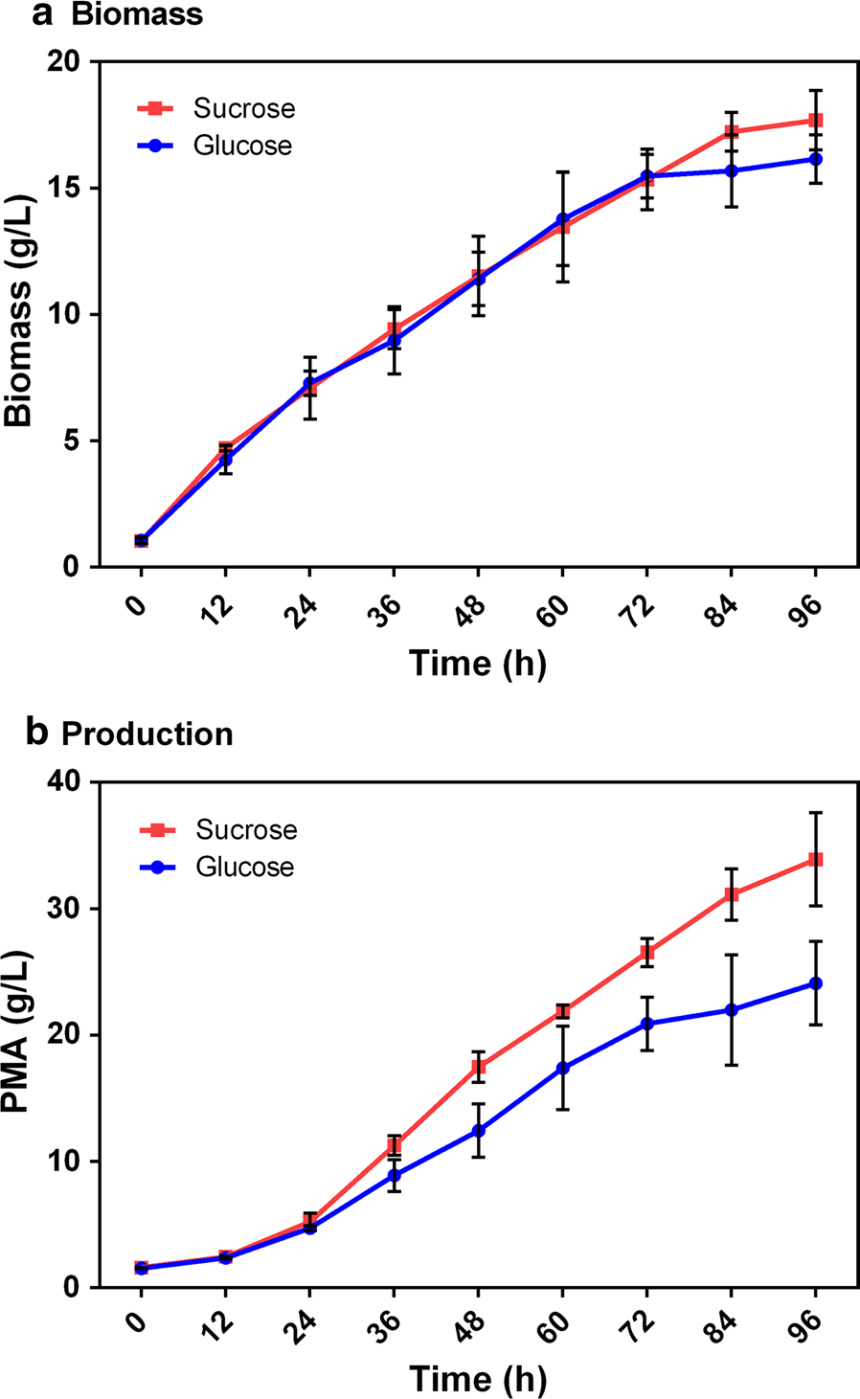
Batch fermentation of PMA production from glucose or sucrose in shake-flask culture. **a** Biomass and **b** PMA production. Glucose: line with blue circles; sucrose: line with red squares. The values shown represent the means of three independent experiments, and the error bars represent standard deviations of three values

Sugarcane molasses is the main by-product of sugar production, and mainly comprises sucrose and some glucose and fructose. In this study, fermentation with different carbon sources in shake-flask culture was investigated (Fig. 1). Of the carbon sources used, sucrose and fructose showed superior PMA production of 28.30 ± 2.58 and 22.58 ± 1.08 g/L, respectively, which was higher than that (20.38 ± 2.32 g/L) from glucose as the carbon source. Moreover, five-carbon sugar (arabinose and xylose) fermentation was also superior to that with glucose for PMA production. In our previous study, a novel PMA-producing strain, *A. pullulans* YJ6-11, was also found to utilize xylose as a superior carbon source compared with glucose [33]. The fermentation kinetics of sucrose and glucose in shake-flask culture are shown in Fig. 2. A total of 33.91 ± 3.70 g/L PMA (~ 38.98 g/L MA after hydrolysis) were produced from ~ 90 g/L sucrose in 96 h via batch fermentation; this was increased by 40.7% compared with that of glucose (24.10 ± 3.30 g/L). These results showed that sucrose present in sugarcane molasses was the most suitable sugar for PMA biosynthesis. However, the underlying factors responsible for the differences between sucrose and glucose were still unclear.

### Analysis of key metabolites and pathways associated with PMA biosynthesis

**Fig. 3:**
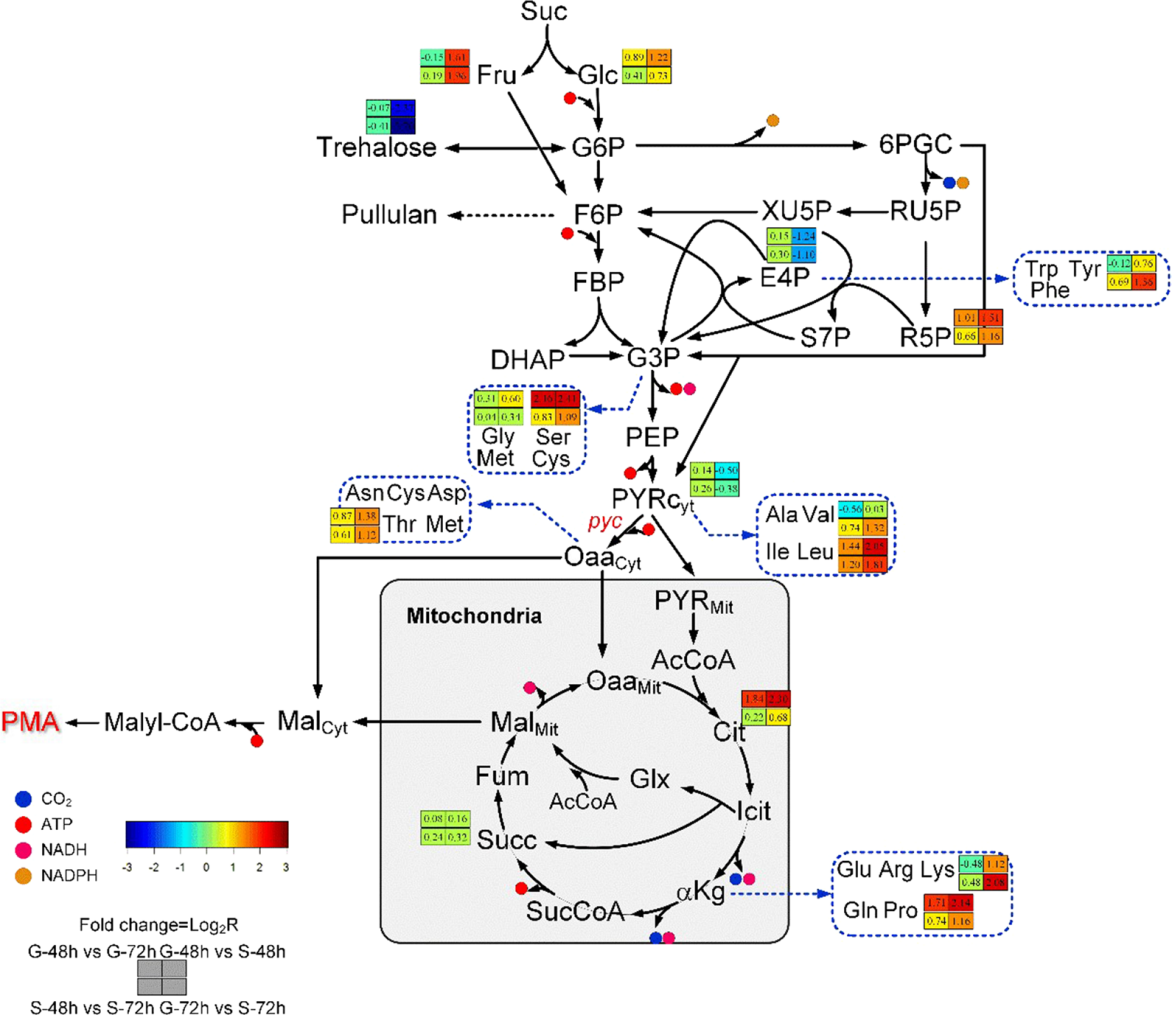
Key metabolite changes in PMA biosynthesis based on comparative metabolome analysis. *Suc* sucrose, *Glc* glucose, *Fru* fructose, *G6P* glucose 6-phosphate, *F6P* fructose-6-phosphate, *FBP* fructose 1,6-bisphosphate, *DHAP* dihydroxyacetone phosphate, *G3P* glyceraldehyde 3-phosphate, *6PGC* gluconate 6-phosphate, *RU5P* ribulose 5-phosphate, *XU5P* xylulose 5-phosphate, *R5P* ribose 5-phosphate, *E4P* erythrose 4-phosphate, *S7P* sedoheptulose 7-phosphate, *PEP* phosphoenolpyruvate, *PYR* pyruvate, *OAA* oxaloacetic acid, *AcCoA* acetyl-CoA, *Cit* citrate, *Icit* isocitrate, *αKg* α-oxoglutarate, *SucCoA* succinyl-CoaA, *Succ* succinate, *Fum* fumarate, *Mal* malate, *Glx* glyoxylate, *Trp* tryptophan, *Tyr* tyrosine, *Phe* phenylalanine, *Gly* glycine, *Ser* serine, *Met* methionine, *Cys* cysteine, *Asn* asparagine, *Asp* aspartic acid, *Thr* threonine, *Ala* alanine, *Val* valine, *Ile* isoleucine, *Leu* leucine, *Glu* glutamic acid, *Arg* arginine, *Lys* lysine, *Gln* glutamine, *Pro* proline. The content of metabolites in *A. pullulans* CTCC2012223 cultured in glucose for 48 h was used as control and defined as 1.00; the contents of the other samples were used to calculate the fold change with the following formula: fold change = log_2_(*R*), *R* = *B*/*A*, *A* is the control, and *B* is the treated sample

To explain the differences in metabolites of *A. pullulans*, the metabolic states of sucrose- and glucose-based fermentation were compared by metabolomics analysis. As shown in Additional file 2: Table S2, in total, 81 intracellular metabolites were identified and divided into eight categories. These groups mainly included organic acids, amino acids, and sugar metabolism that contained 21, 15, and 14 metabolites, respectively. Among these metabolites, compared with culture at 48 h, 13 and 3 differential metabolites were increased at 72 h in glucose and sucrose, respectively. 27 and 30 differentially abundant metabolites from glucose versus sucrose after culture for 48 and 72 h, respectively, were validated. Of these metabolites, both at 48 and 72 h, 16 in sucrose fermentation, including fructose, ribose, glycolic acid, oleic acid, and heptanoic acid, were higher than that of glucose (Additional files 3 and 4: Figure S1 and Table S3). These results indicated that the differences of metabolites were varied in the different sugar fermentation. Moreover, compared with glucose fermentation at 48 h, 13 differential metabolites were increased at 72 h. Notably, at 72 h, citric acid was increased by 1.84-fold, whereas other intermediate metabolites associated with the TCA cycle (such as pyruvic acid, succinic acid, and fumaric acid) did not differ significantly at 48 h (Additional file 4: Table S3). However, at 72 h, MA was decreased by 1.04-fold compared with that at 48 h, which was negative correlated with citric acid (Fig. 3). This may induce a decrease in PMA biosynthesis, because accumulation of citric acid resulted in carbon flux that was not efficiently channeled to the objective product. Pyruvate is a key node in central carbon metabolism and plays a major role in pathways related to organic acid synthesis [34]. The carbon flux can be channeled into different products through pyruvate, including succinate [35], lactate [36], and malate [37]. The reductive TCA cycle has been identified as the most efficient pathway for many products, such as succinate [38] and malate [37]. Thus, the accumulation of citric acid also indicated that metabolic flux could not effectively direct pyruvate to PMA synthesis or the high-efficiency PMA synthesis route in native metabolic regulation. Therefore, the enhanced carboxylation of pyruvate to oxaloacetate, catalyzed by pyruvate carboxylase, may be an important target for improving PMA synthesis.

In addition, pyruvate is also a major component in pathways related to amino acid metabolism. Amino acids are crucial to microorganism metabolism and have roles in synthesis of proteins (e.g., leucine, threonine, and phenylalanine), regulation of gene expression (e.g., arginine and leucine), osmoregulation (e.g., citrulline and proline), control of enzyme activity (e.g., alanine and leucine), transamination (e.g., aspartate, alanine, and glutamate), and ammonia detoxification (e.g., citrulline, arginine, and glutamate) [39]. As shown in Fig. 3, amino acids present at higher levels, particularly leucine and proline, could be directly or indirectly transformed from pyruvate and α-oxoglutarate [40]. Most amino acids were observed at higher levels in sucrose than in glucose because of the abundant precursors in the glycolytic pathway and TCA cycle. The redox balance is maintained by modifying the distribution of the metabolic flux of pyruvate [41].

In addition, when sucrose was used as the carbon source, relative concentration of trehalose was significantly decreased 2.37- and 2.70-fold at 48 and 72 h, respectively, compared with that when glucose was used as the carbon source (Additional file 4: Table S3). Accumulation of trehalose in yeasts has been shown to be an important mechanism mediating tolerance against adverse stress conditions, and trehalose functions as a reserve carbohydrate that can be synthesized when the exogenous energy exceeds the cellular needs for growth and biosynthesis [42, 43]. These results suggested that glucose as a rapidly metabolizable carbon source can induce a regulatory response (e.g., osmotic stress) to restore excess exogenous energy, which would cause some of the carbon source to be used for other processes, not PMA synthesis, thereby decreasing PMA titers.

### In silico analysis of a genome-scale metabolic model

**Fig. 4:**
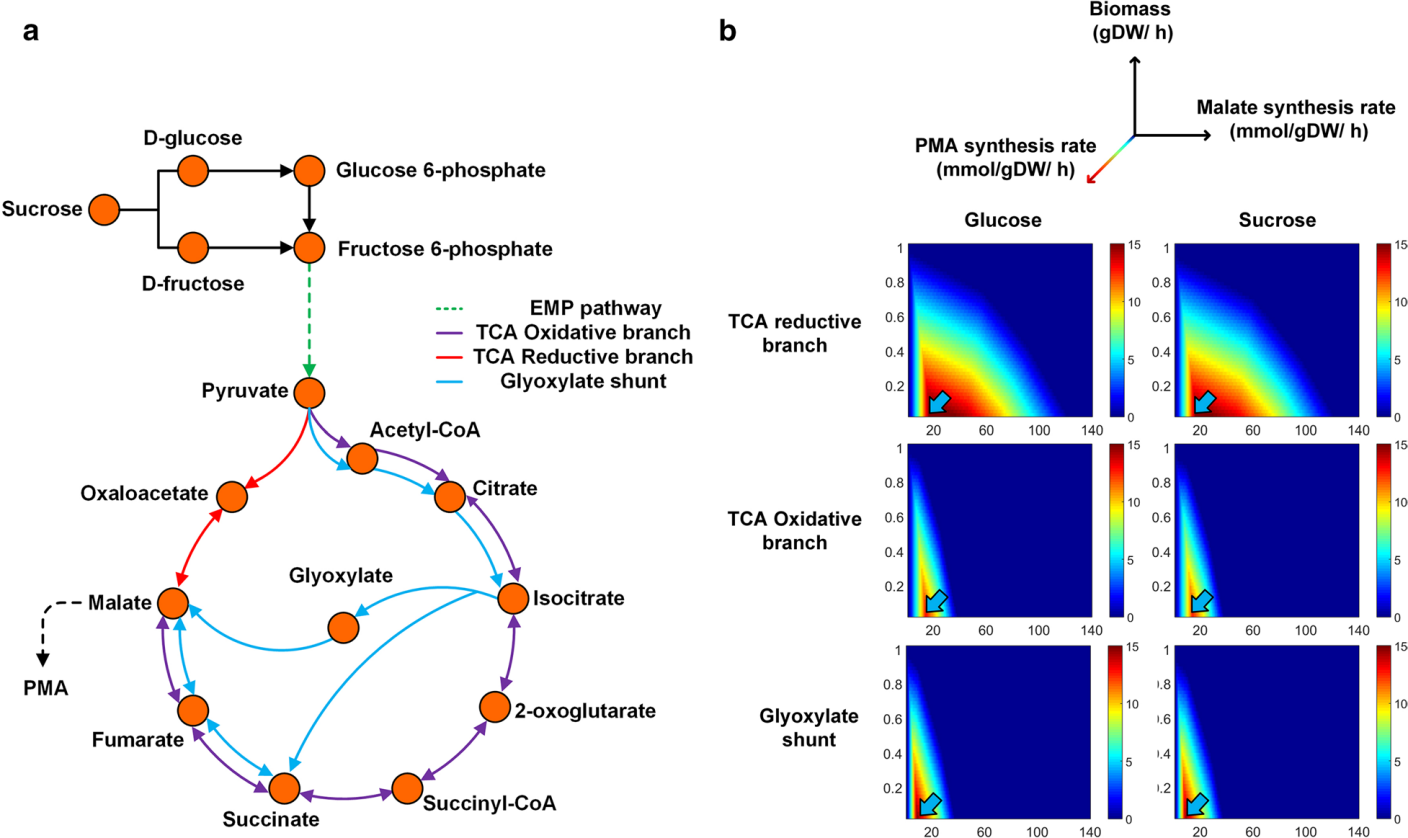
Evaluation of malate synthesis route for PMA production based on genome-scale metabolic simulation. **a** Schematic of simulation analysis in different malate synthesis route. **b** *X*- and *Y*-axes show malate synthesis route flux (mmol/gDW/h) and PMA production rate (mmol/gDW/h), respectively. The *Z*-axis represents the cell growth rate (gDW/h). The color gradient represents the value according to the scale shown in the box

PMA is synthesized from MA, which is an intermediate in the TCA cycle in aerobic metabolism. Genome-scale flux sensitivity analyses were performed to determine the most effective route for maximizing PMA synthesis. Three metabolic routes involved in MA synthesis, including oxidative branches of the TCA cycle, reductive branches of the TCA cycle, and glyoxylate shunt, were simulated using the genome-scale metabolic model (*i*ZX637). As shown in Fig. 4, the optimal PMA synthesis rate, using glucose or sucrose as the carbon source, reached 15 mmol/gDW/h (indicated by an arrow), increasing the reductive TCA malate synthesis rate to 15 mmol/gDW/h and the biomass growth rate to 0 mmol/gDW/h. When the carbon flux turned to oxidative branches of the TCA cycle and the glyoxylate shunt, the optimal PMA synthesis rates reached only as high as 12.89 mmol/gDW/h (sucrose as a carbon source: 12.58 mmol/gDW/h) and 14.67 mmol/gDW/h (sucrose as a carbon source: 14.33 mmol/gDW/h) with corresponding malate synthesis rates of 13 and 8 mmol/gD/h, respectively. In addition, the malate synthesis rate of the corresponding route constantly increased, resulting in a negative influence on the optimal PMA synthesis rate. When the malate synthesis rates of the reductive TCA route, oxidative TCA route, and glyoxylate shunt reached 120, 37, and 37 mmol/gDW/h, respectively, the PMA synthesis rate and biomass growth rate were reduced to 0 mmol/gDW/h (glucose or sucrose as a carbon source). Furthermore, the PMA synthesis rate decreased gradually as the biomass synthesis rate increased for all simulation results. These results showed that PMA and biomass synthesis showed a competitive relationship. However, in the reductive TCA route, biomass synthesis could be maintained with a relatively high PMA synthesis rate compared with the other two routes, regardless of whether glucose or sucrose was used as the carbon source. In our previous study, we found that high levels of the nitrogen source favored cell growth but decreased PMA synthesis [44]. Therefore, the efficiency of the PMA synthesis route should balance cell growth and PMA formation. In our previous study, in silico analysis of the carbon flux distribution and changes in PMA synthesis rates showed that, in the context of a high PMA synthesis rate, a large amount of carbon flux was transformed into pyruvate and channeled into the reductive TCA route by pyruvate carboxylase [27]. These in silico results showed that the reductive TCA route, as the optimal PMA synthesis route, showed a relatively high optimal PMA synthesis rate and mitigated the competitive relationship between PMA and biomass synthesis. In addition, the simulation results also showed that the reductive TCA route required a relatively high route flux (the reductive TCA malate synthesis rate to 15 mmol/gDW/h), yielding a high PMA synthesis rate (the optimal PMA synthesis rate to 15 mmol/gDW/h). Therefore, pyruvate carboxylase (*pyc*) in the reductive TCA route was selected as the target to be overexpressed to improve PMA yield and productivity.

Moreover, according to the simulation result from sucrose and glucose as carbon sources, sucrose was not dominant compared with glucose as a carbon source for the optimal PMA synthesis rate and cell growth by *A. pullulans* strains in the conversion efficiency of metabolic pathways. This could be explained by the observations that the reaction of sucrose hydrolysis exhibited no energy consumption or carbon loss in silico.

### Overexpression of the *pyc* gene in *A. pullulans*

**Fig. 5:**
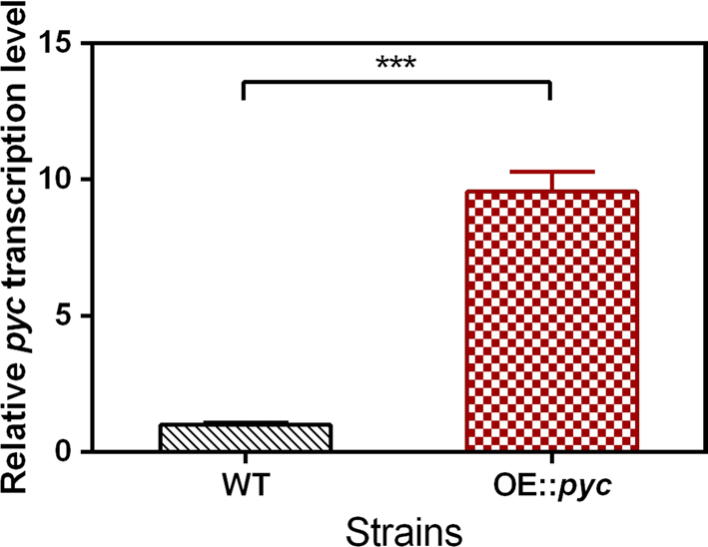
Expression level of the *pyc* gene in the engineered strain E10. Each column was calculated with three parallel experiments. ***P < 0.001 versus the wild-type strain

To evaluate the effects of the *pyc* gene on PMA biosynthesis, the endogenous *pyc* gene was amplified using the genomic DNA of *A. pullulans* as a template (Additional file 3: Figure S2). The P*gpdA* promoter of *Aspergillus nidulans*, which directs the constitutively high expression of the glyceraldehyde-3-phosphate dehydrogenase (*gpd*) gene, can be used to induce high levels of constitutive expression of genes of interest or marker genes and to drive the overexpression of various genes in fungi [45–47]. Therefore, the P*gpdA* promoter of *A. nidulans* was used to constitute the *pyc* cassette by cloning into pBARGPE1 (Additional file 3: Figure S3). In our previous study, we developed a simple and efficient system for genetic transformation of *A. pullulans* using *A. tumefaciens* [30]. Therefore, *A. tumefaciens* carrying the pK2-*hyg*-*pyc* binary plasmid was used to transform *A. pullulans*. Because ATMT-mediated integration is random, five different ATMT-derived clones were tested for PMA production in shake-flask culture. The PMA titers of five clones compared with the wild-type strain were improved from 3.5 to 7.1% (Additional file 3: Figure S4). The differences among mutants were due to random integration, causing interruption of gene function by insertion of T-DNA, and were not caused by intrinsic factors [30]. This result indicated that the increased PMA titer was mainly dependent on *pyc* gene overexpression. Thus, we selected the highest PMA producer (E10 strain; as called FJ-PYC) for further analysis of the sequences flanking the T-DNA in the genome. As shown in Additional file 3: Figure S5, the hygromycin B resistance cassette and overexpression *pyc* cassette were inserted into the noncoding regions of g4940.t1 and g4941.t1 on the *A. pullulans* genome. The insertions did not disrupt the g4940.t1 and g4941.t1 open reading frames. Furthermore, we found that the expression level of the *pyc* gene in strain E-10 was increased by 9.5-fold compared with that in the wild-type strain (Fig. 5). These results also revealed that the promoter P*gpdA* was an effective tool for regulating gene expression in *A. pullulans*.

**Fig. 6:**
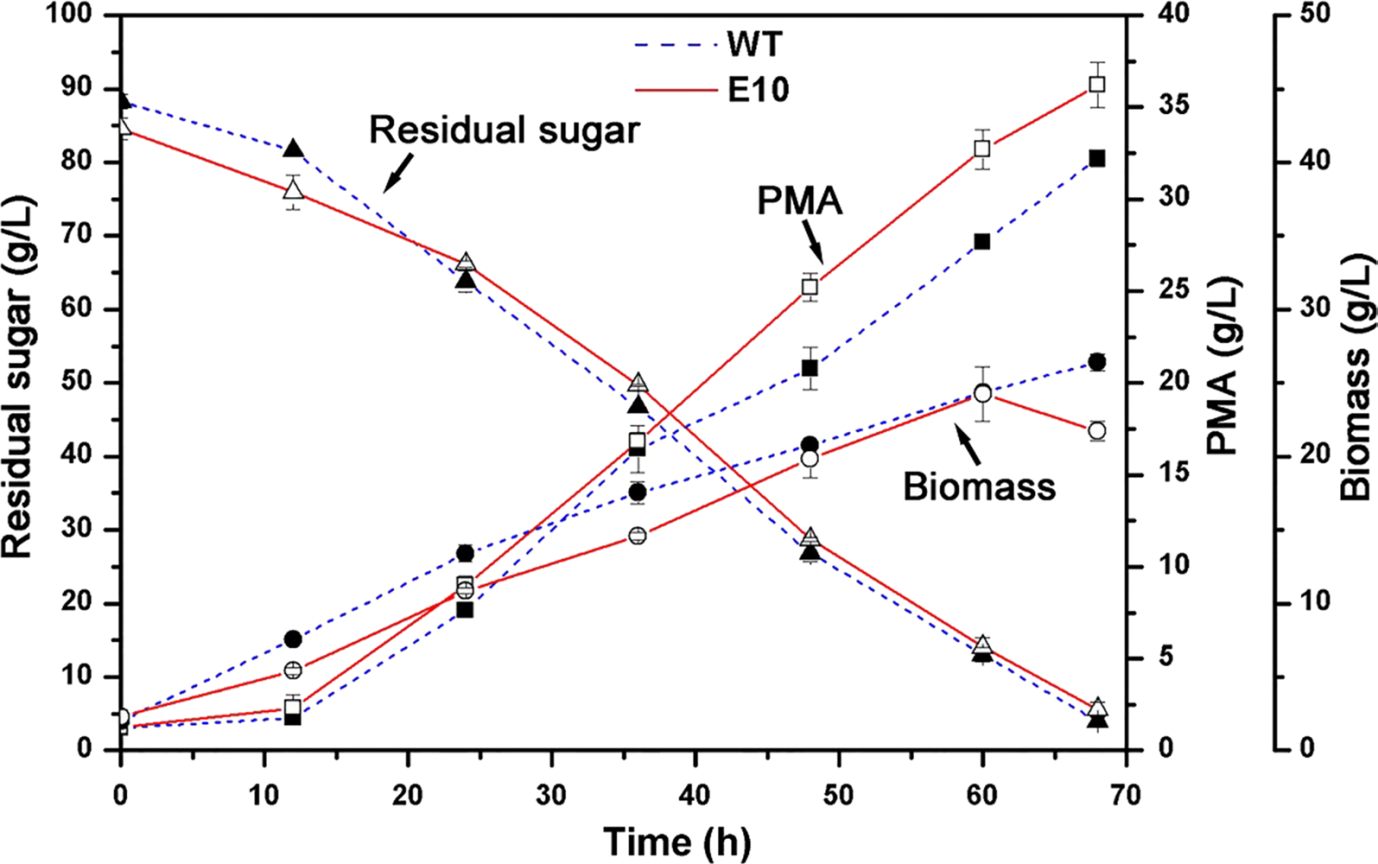
Batch fermentation of PMA production with the engineered strain E10 in a stirred-tank fermentor. All the data were derived from two independent experiments

Subsequently, the engineered strain E10 was tested with glucose as the carbon source in a 5-L stirred-tank fermentor. As shown in Fig. 6, about 36.2 g/L of PMA was produced via batch fermentation, with a PMA productivity of 0.53 g/L h, which was increased by 15.1 and 12.7% compared with the control, respectively. This result indicated that overexpression of the *pyc* gene could improve the production of PMA, which was consistent with the results of in silico simulation showing that strengthening of the reductive TCA route resulted in increased carbon flux toward PMA synthesis.

### Batch and fed-batch fermentation with sugarcane molasses

**Fig. 7:**
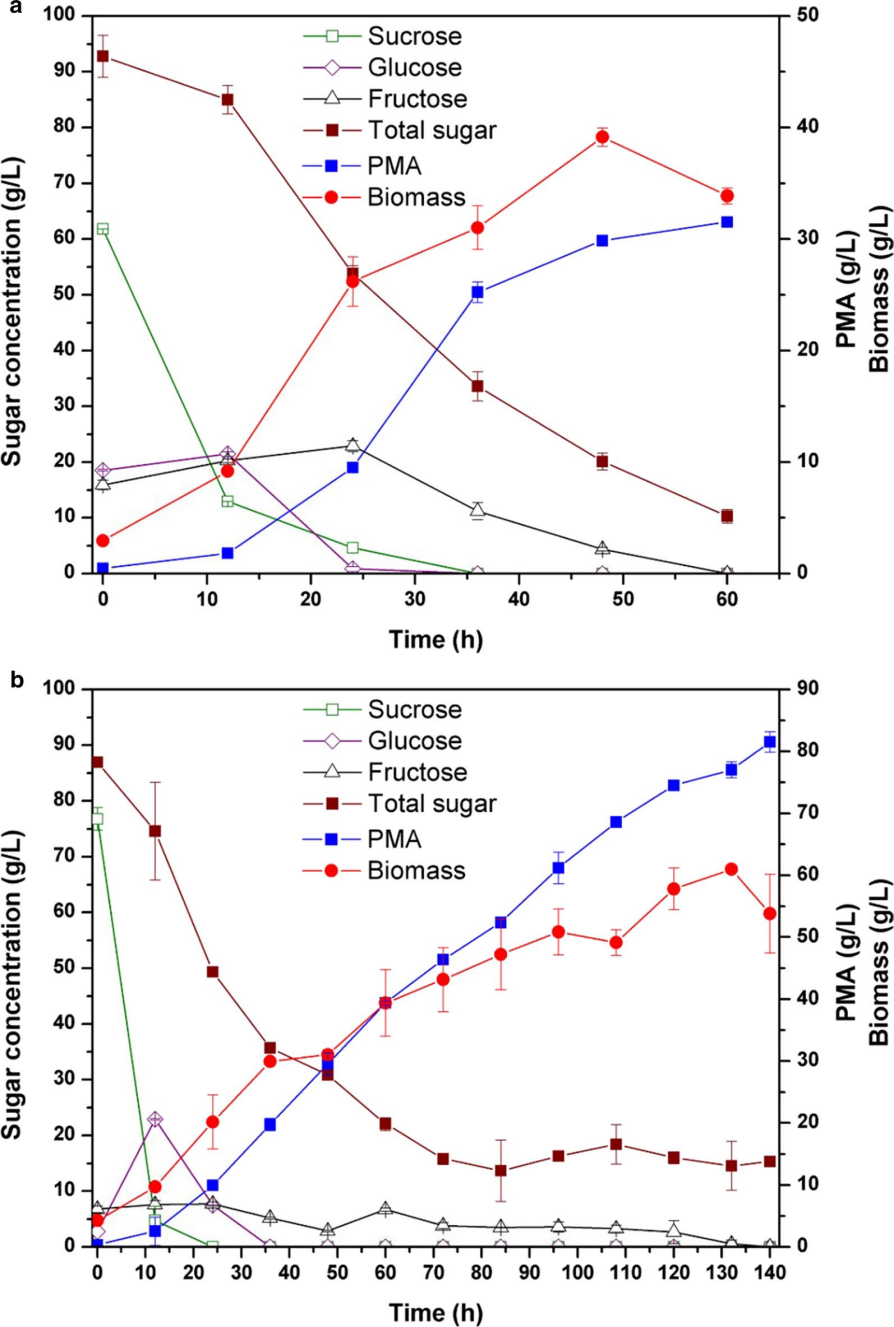
Fermentation kinetics of PMA production from sugarcane molasses in a stirred-tank fermentor. **a** Batch fermentation; **b** fed-batch fermentation. PMA production is reported as malic acid content after acid hydrolysis of PMA. All the data were derived from two independent experiments

Sugarcane molasses is a low-cost by-product of sugar production and mainly contains sucrose, with some fructose and glucose. To develop an economical fermentation process, sugarcane molasses without any pretreatment or nutrient supplementation was employed for PMA production by FJ-PYC strain in a 5-L stirred-tank fermentor. As shown in Fig. 7a, during the early fermentation stage, sucrose was rapidly consumed and transformed into glucose and fructose, and glucose was then used for cell growth and PMA synthesis compared with fructose. This phenomenon, called glucose-mediated carbon catabolite repression (CCR), is widely observed in bacteria and yeasts [48–50]. About 31.5-g/L PMA (36.5-g/L MA after hydrolysis) was produced from ~ 95-g/L mixed sugar (61.8-g/L sucrose, 18.5-g/L glucose, and 15.3-g/L fructose from sugarcane molasses) in 60 h via batch fermentation, with an MA yield of 0.44 g/g. Moreover, without adding any other media, the productivity of PMA (0.53 g/L h) was the same as that from glucose fermentation. In fed-batch fermentation (Fig. 7b), a high final PMA titer of 81.5 g/L (94.2-g/L MA after hydrolysis) was achieved in 140 h with a corresponding MA yield of 0.62 g/g and productivity of 0.67 g/L h. As shown in Table 1, compared with other renewable feedstocks, PMA fermentation from sugarcane molasses by strain FJ-PYC resulted in the highest titer and productivity. Nevertheless, the titer and productivity could be further increased by continuous fed operation, because the PMA synthesis rate maintained a high speed. In addition, the fed-batch fermentation with FJ-PYC resulted in an overall MA yield of 0.62 g/g from sugarcane molasses without nitrogen supplementation, which was the same as the highest yield from sugarcane juice [49]. However, sugarcane juice contained only ~ 15% reducing sugars and relatively fewer inhibitors, requiring it to be condensed for high-sugar PMA fermentation and dramatically decreasing its economic efficiency. These results indicated that the strain FJ-PYC exhibited extremely high adaptation and tolerance against sugarcane molasses.

**Table 1:** PMA production from various biomass substrates by different strains of *A. pullulans*

Microorganism	Substrates	Nitrogen sources	Operating mode	PMA^a^ (g/L)	Malic acid (g/L)	Productivity^a^ (g/L h)	Yield^a^ (g/g)	References
NRRL 50383	Corn fiber	Peptone and yeast extract	Batch	10.1	11.7^b^	0.07	–	[13]
	Wheat straw	Peptone and yeast extract	Batch	23.5	27.1^b^	0.16	–	
ZX-10	Soybean hull hydrolysate	Corn steep liquor	Fed-batch	27.2	31.3	0.48	0.42	[12]
	Soy molasses	–	Fed-batch	62.6	71.9	0.29	0.69	
	Sugarcane juice	–	Batch	52.6	60.8	0.32	0.62	[49]
CCTCCM2012223	Hydrolysate of raw sweet potato	NH_4_NO_3_	Batch	29.6	33.6	0.28	0.31	[9]
	Hydrolysate of raw sweet potato	NH_4_NO_3_	Fed-batch	44	49.9	0.31	0.22	
YJ 6-11	Corncob hydrolysate	NH_4_NO_3_	Batch	28.6	32.4	0.45	0.41	[33]
FJ-PYC	Sugarcane molasses	–	Batch	31.5	36.5	0.61	0.44	This study
		–	Fed-batch	81.5	94.2	0.67	0.62	

-, none or not reported

^a^To facilitate comparisons, PMA yield and productivity were based on the malic acid that can be released from PMA after hydrolysis, PMA (g/L) = 0.87 malic acid (g/L)

^b^Calculated from data in this study

## Conclusions

In this study, different carbon sources were evaluated, and sugarcane molasses was assessed as a potential feedstock for economic PMA and MA production. Among the different carbon sources examined in this study, sucrose was found to be the optimal carbon source for PMA biosynthesis. Metabolomics analysis of sucrose- and glucose-based fermentation identified a total of 81 intracellular metabolites and showed that pyruvate from the glycolysis pathway may be a key metabolite affecting PMA synthesis. In silico analysis of a genome-scale metabolic model (*i*ZX637) verified that the *pyc* gene via the reductive TCA cycle was a target affecting PMA synthesis. Consistent with this, overexpression of the *pyc* gene in *A. pullulans* strain FJ-PYC increased the PMA titer by 15.1% compared with the control.

Moreover, sugarcane molasses could be directly utilized by this recombinant strain without any pretreatment or nutrient supplementation, producing 31.5-g/L PMA (36.5-g/L MA) with a high-level PMA productivity of 0.53 g/L h in batch fermentation. In fed-batch fermentation, compared with the other renewable feedstock, the highest final PMA titer of 81.5 g/L (94.2-g/L MA after hydrolysis) was achieved in 140 h, with a corresponding MA yield of 0.62 g/g and productivity of 0.67 g/L h. This study demonstrated the great potential of sugarcane molasses for the economical production of PMA and MA on the industrial scale.

## Additional files

**Additional file 1: Table S1.** List of strains, plasmids, and primers.

**Additional file 2: Table S2.** Identified metabolites by comparison of mass spectra.

**Additional file 3: Figure S1.** The statistical numbers of differential metabolites between groups. Red bars represent the numbers of relative concentration increased metabolites and blue bars represent the numbers of relative concentration decreased metabolites. **Figure S2.** PCR amplification of the endogenous *pyc* gene using the genomic DNA of *A. pullulans*. **Figure S3.** Construction of the *pyc* cassette by cloning into the plasmid pBARGPE1 with the P*gpdA* promoter. **Figure S4.** Batch fermentation of different ATMT-derived clones with glucose as carbon source in shake flask. **Figure S5.** Analysis of sequences flanking to T-DNA in genome of the strain E10.

**Additional file 4: Table S3.** The changes of differential metabolites in glucose- and sucrose-based fermentation.

## Abbreviations

PMA: polymalic acid
MA: l-malic acid
*pyc*: pyruvate carboxylase gene
TCA: tricarboxylicacid
GEM: genome scale metabolic model
HPLC: high-performance liquid chromatography
BSTFA: bis(trimethylsilyl) trifluoroacetamide
GC–MS: gas chromatography–mass spectrometry
EI: electron ionization
NIST: National Institution of Standards and Technology
GM: Golm Metabolome
PCA: principal component analysis
OPLS-DA: orthogonal-partial least squares-discriminant analysis
FDR: false discovery rate
P*gpdA*: gpdA promoter
T*trpC*: trpC terminator
ATMT: *A. tumefaciens*-mediated transformation
PCR: polymerase chain reaction
*gpd*: glyceraldehyde-3-phosphate dehydrogenase gene
CCR: carbon catabolite repression
