# Supplementary material for: Metabolome- and genome-scale model analyses for engineering of Aureobasidium pullulans to enhance polymalic acid and malic acid production from sugarcane molasses
Source: Biotechnol Biofuels. 2018 Apr 4;11:94. doi: 10.1186/s13068-018-1099-7 (PMC5883625; doi:10.1186/s13068-018-1099-7)
Supplement: Supplementary file 1 — Additional file 1: Table S1. List of strains, plasmids, and primers. [file 13068_2018_1099_MOESM1_ESM.docx]

**Table S1.** List of strains, plasmids, and primers

| Strain/plasmid/primer | Relevant characteristics | Reference/source |
| --- | --- | --- |
| **Strains** |  |  |
| *A. pullulans* CCTCCM 2012223 | Wild-type strain | CCTCC |
| *A. pullulans* FJ-PYC | *pyc*-overexpressing strain | This work |
| *A. tumefaciens* | Used for transformation of *A. pullulans* |  |
| *E. coli* DH5α | Host cells for plasmids preparation | Tiangen |
| **Plasmids** |  |  |
| pK2-P*trpC*-*hyg*-T*trpC* | *KanR* for bacteria, *HygR* for fungus, binary plasmid, for *Agrobacterium*-mediated transformation |  |
| pK2-*hyg*-*pyc* | *pyc*-overexpressing plasmid, binary plasmid for *Agrobacterium*-mediated transformation | This work |
| pBARGPE1 | AmpR for bacteria, P*gpdA*: promoter; T*trpC*: terminator; bar: phosphinothricin acetyltransferase gene for a selection marker; fungal expression plasmid |  |
| **Primer** |  |  |
| Sma I-*pyc*-S | 5′-atgcccgggatgatgtcggacattgaagctct-3′ | This work |
| Mun I-*pyc*-A | 5′-gcgcaattggcgctatttgacgatcttgcaaatgagg-3′ |  |
| PgpdA.*pyc*.T*trpC*-S | 5′-attacgaattccccgggggatctggtgcactctcagtacaatc-3′ | This work |
| P*gpdA*.*pyc*.T*trpC*-A | 5′-ggagcatacccaacactagtggatccccatctcataaataacgtcatg-3′ |  |
| *β-actin*-S | 5′-gaagtgcgatgtcgatgtcaga-3′ | This work |
| *β-actin*-A | 5′-ggagcaagggcggtgatt-3′ |  |
| *pyc*-S | 5′-cagtgggcacagacaaagaagg-3′ | This work |
| *pyc*-A | 5′-accctcgaagaactcaaggacag-3′ |  |
| P*trpC*-TSP-1 | 5′-tgttatccgctcacaattcca -3′ | This work |
| P*trpC*-TSP-2 | 5′-gctcactgcccgctttcca-3′ |  |
| P*trpC*-TSP-3 | 5′-gagaggcggtttgcgtattgg-3′ |  |
