# Supplementary material for: Metabolome- and genome-scale model analyses for engineering of Aureobasidium pullulans to enhance polymalic acid and malic acid production from sugarcane molasses
Source: Biotechnol Biofuels. 2018 Apr 4;11:94. doi: 10.1186/s13068-018-1099-7 (PMC5883625; doi:10.1186/s13068-018-1099-7)
Supplement: Supplementary file 3 — Additional file 3: Figure S1. The statistical numbers of differential metabolites between groups. Red bars represent the numbers of relative concentration increased metabolites and blue bars represent the numbers of relative concentration decreased metabolites. Figure S2. PCR amplification of the endogenous pyc gene using the genomic DNA of A. pullulans. Figure S3. Construction of the pyc cassette by cloning into the plasmid pBARGPE1 with the PgpdA promoter. Figure S4. Batch fermentation of different ATMT-derived clones with glucose as carbon source in shake flask. Figure S5. Analysis of sequences flanking to T-DNA in genome of the strain E10. [file 13068_2018_1099_MOESM3_ESM.doc]

**Additional file 3**

**Figure S1.** The statistical numbers of differential metabolites between groups. Red bars represent the numbers of relative concentration increased metabolites and blue bars represent the numbers of relative concentration decreased metabolites.

**Figure S2**. PCR amplification of the endogenous *pyc* gene using the genomic DNA of *A. pullulans*.

**Figure S3**. Construction of the *pyc* cassette by cloning into the plasmid pBARGPE1with the P*gpdA* promoter

**Figure S4**. Batch fermentation of different ATMT-derived clones with glucose as carbon source in shake flask.

**Figure S5**. Analysis of sequences flanking to T-DNA in genome of the strain E10.

**
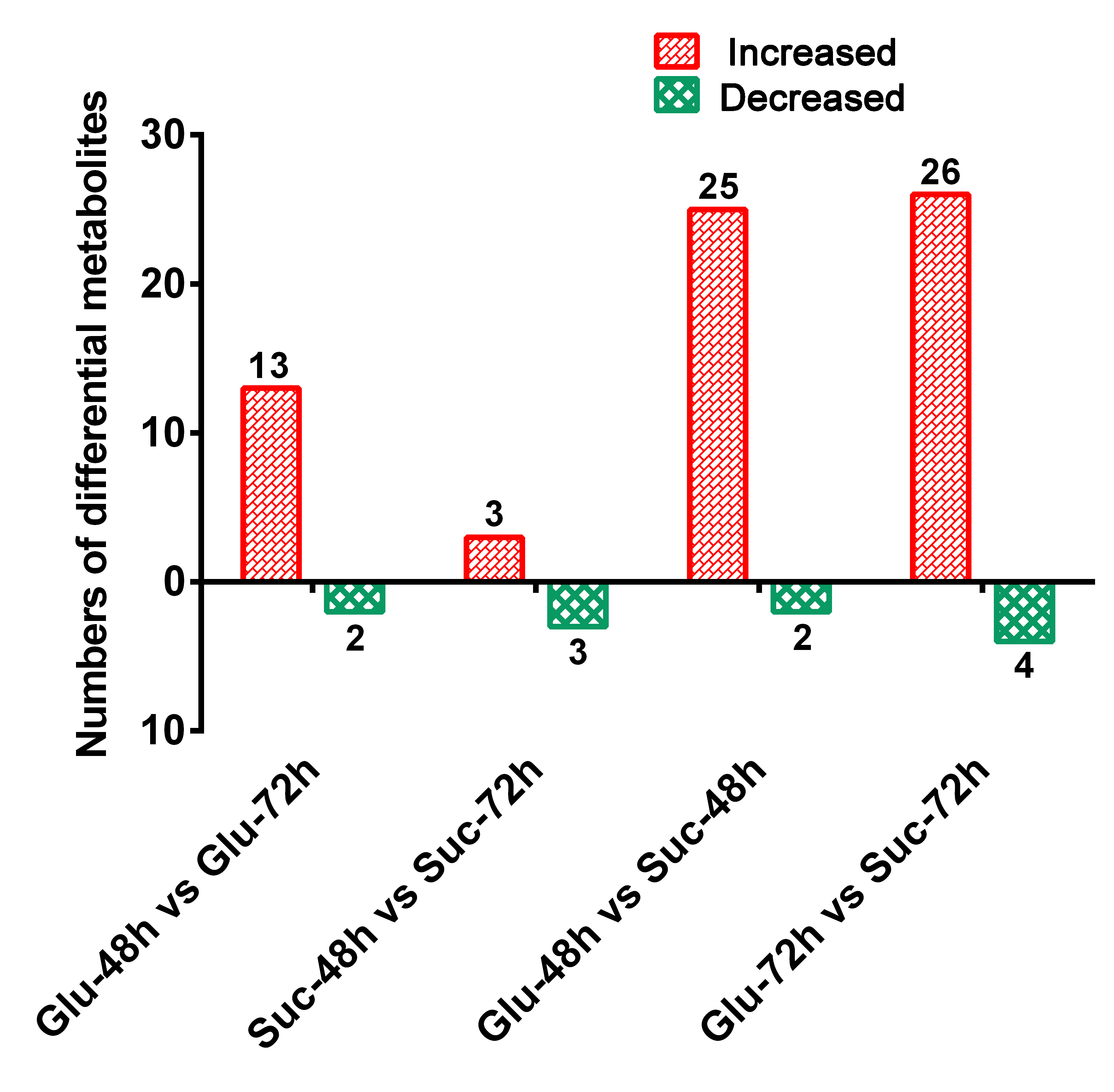
**

**Figure S1**


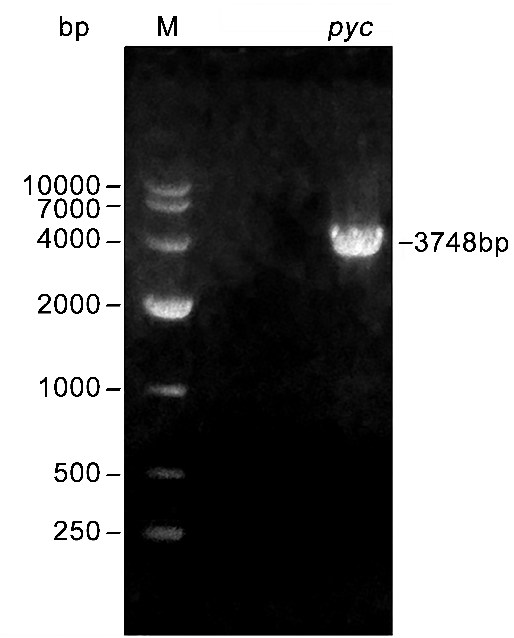


**Figure S2**


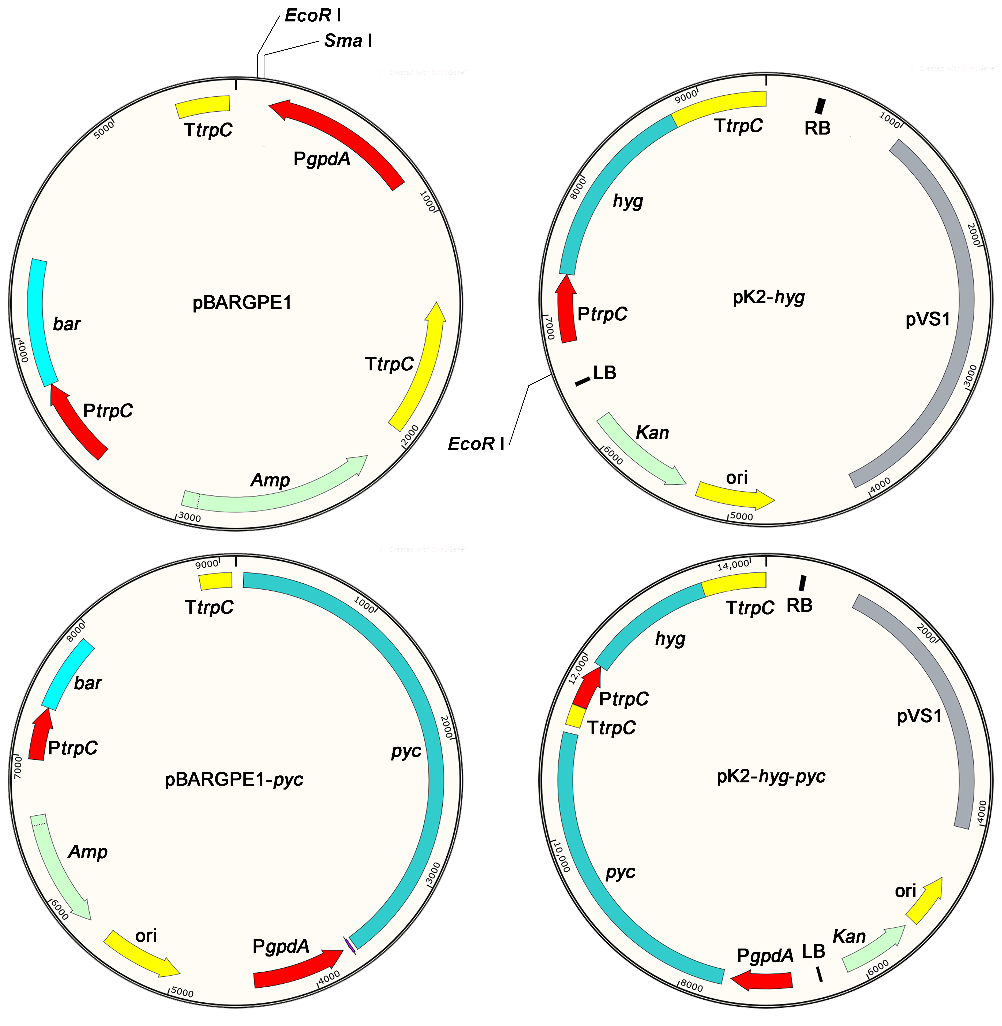


**Figure S3**


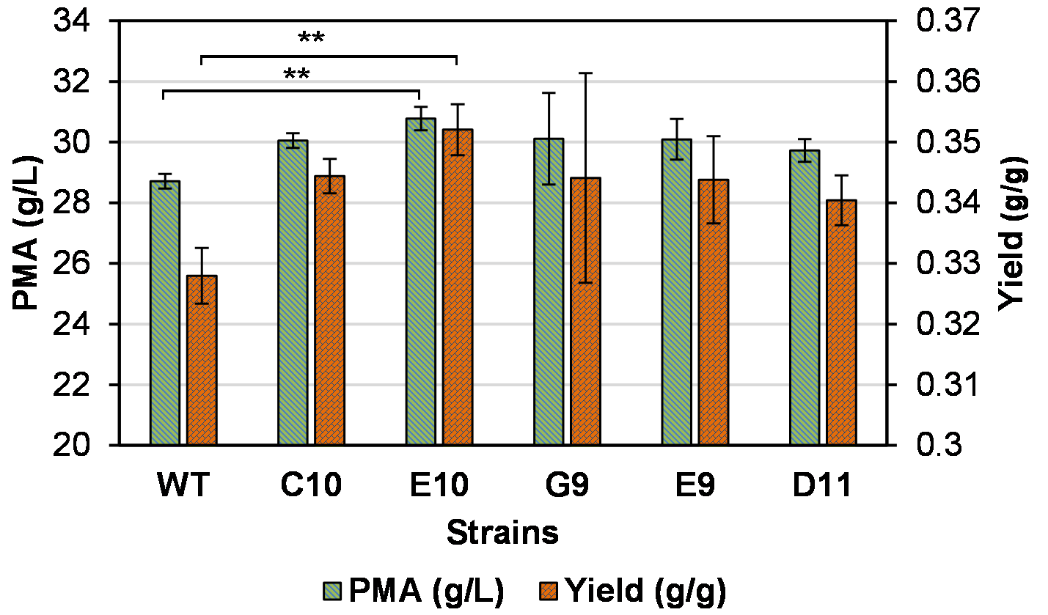


Figure S4


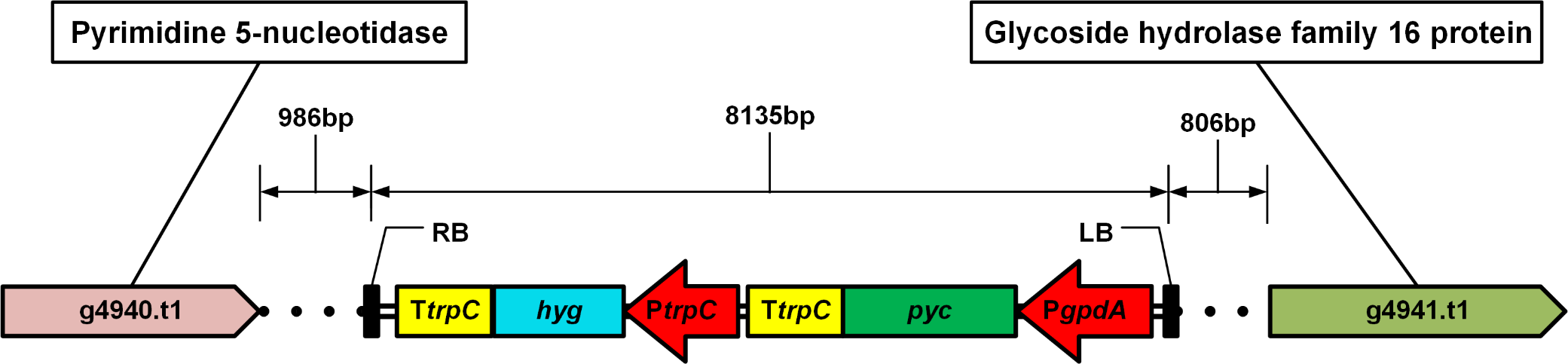


**Figure S5**
